# Coronary versus carotid blood flow and coronary perfusion pressure in a pig model of prolonged cardiac arrest treated by different modes of venoarterial ECMO and intraaortic balloon counterpulsation

**DOI:** 10.1186/cc11254

**Published:** 2012-03-16

**Authors:** Jan Bělohlávek, Mikuláš Mlček, Michal Huptych, Tomáš Svoboda, Štěpán Havránek, Petr Ošt'ádal, Tomáš Bouček, Tomáš Kovárník, František Mlejnský, Vratislav Mrázek, Marek Bělohlávek, Michael Aschermann, Aleš Linhart, Otomar Kittnar

**Affiliations:** 1 2nd Department of Medicine - Department of Cardiovascular Medicine, First Faculty of Medicine, Charles University in Prague and General University Hospital in Prague, U Nemocnice 2, Prague 2, 128 00, Czech Republic; 2 Department of Physiology, 1st Faculty of Medicine, Charles University in Prague, Albertov 5, Prague 2, 128 00, Czech Republic; 3 BioDat Research Group, Department of Cybernetics, Faculty of Electrical Engineering, Czech Technical University in Prague, Karlovo namesti 13, Prague 2, 121 35, Czech Republic; 4 Department of Cardiology, Na Homolce Hospital, Roentgenova 2/37, Prague 5, 150 30, Czech Republic; 5 2nd Department of Surgery, Cardiovascular Surgery, First Faculty of Medicine, Charles University in Prague and General University Hospital in Prague, U Nemocnice 2, Prague 2, 128 00, Czech Republic; 6 Translational Ultrasound Research Laboratory, Division of Cardiovascular Diseases, Mayo Clinic Arizona, Scottsdale, AZ, USA

**Keywords:** cardiac arrest, extracorporeal membrane oxygenation, coronary flow velocity, carotid flow velocity

## Abstract

**Introduction:**

Extracorporeal membrane oxygenation (ECMO) is increasingly used in cardiac arrest (CA). Adequacy of carotid and coronary blood flows (CaBF, CoBF) and coronary perfusion pressure (CoPP) in ECMO treated CA is not well established. This study compares femoro-femoral (FF) to femoro-subclavian (FS) ECMO and intraaortic balloon counterpulsation (IABP) contribution based on CaBF, CoBF, CoPP, myocardial and brain oxygenation in experimental CA managed by ECMO.

**Methods:**

In 11 female pigs (50.3 ± 3.4 kg), CA was randomly treated by FF versus FS ECMO ± IABP. Animals under general anesthesia had undergone 15 minutes of ventricular fibrillation (VF) with ECMO flow of 5 to 10 mL/kg/min simulating low-flow CA followed by continued VF with ECMO flow of 100 mL/kg/min. CaBF and CoBF were measured by a Doppler flow wire, cerebral and peripheral oxygenation by near infrared spectroscopy. CoPP, myocardial oxygen metabolism and resuscitability were determined.

**Results:**

CaBF reached values > 80% of baseline in all regimens. CoBF > 80% was reached only by the FF ECMO, 90.0% (66.1, 98.6). Addition of IABP to FF ECMO decreased CoBF to 60.7% (55.1, 86.2) of baseline, *P *= 0.004. FS ECMO produced 70.0% (49.1, 113.2) of baseline CoBF, significantly lower than FF, *P *= 0.039. Addition of IABP to FS did not change the CoBF; however, it provided significantly higher flow, 76.7% (71.9, 111.2) of baseline, compared to FF + IABP, *P *= 0.026. Both brain and peripheral regional oxygen saturations decreased after induction of CA to 23% (15.0, 32.3) and 34% (23.5, 34.0), respectively, and normalized after ECMO institution. For brain saturations, all regimens reached values exceeding 80% of baseline, none of the comparisons between respective treatment approaches differed significantly. After a decline to 15 mmHg (9.5, 20.8) during CA, CoPP gradually rose with time to 68 mmHg (43.3, 84.0), *P = *0 .003, with best recovery on FF ECMO. Resuscitability of the animals was high, both 5 and 60 minutes return of spontaneous circulation occured in eight animals (73%).

**Conclusions:**

In a pig model of CA, both FF and FS ECMO assure adequate brain perfusion and oxygenation. FF ECMO offers better CoBF than FS ECMO. Addition of IABP to FF ECMO worsens CoBF. FF ECMO, more than FS ECMO, increases CoPP over time.

## Introduction

A venoarterial (VA) extracorporeal membrane oxygenation (ECMO) based approach is used for urgent hemodynamic stabilization in patients with profound cardiogenic shock and, increasingly, also in cardiac arrest, both during in-hospital and out-of-hospital cardiac arrest in adults [1-7], in prolonged refractory cardiac arrest [7,8], for in-hospital pediatric cardiac arrest [9,10] and, recently, has even been discussed for out-of hospital "on scene" refractory cardiac arrest management [11]. This approach, also referred to as extracorporeal cardiopulmonary resuscitation (E-CPR) allows perfusion of vital organs during cardiac arrest and provides a time span for diagnosis and therapy [12]. The most demanding vital organs are the brain and the heart, and their adequate oxygenation and perfusion are critical prerequisites for favorable clinical outcomes [13,14]. Additionally, most cardiac arrests, including refractory cases, are caused by either acute coronary or other cardiac events, which may have treatable causes [12]. In clinical practice, a combination of a pulsatile support by intraaortic balloon counterpulsation (IABP) with ECMO is considered beneficial and is used both in ECMO-treated cardiogenic shock and during weaning from extracorporeal support [15]. However, it remains unclear whether coronary and carotid blood flows and coronary perfusion pressure in prolonged cardiac arrest are adequate when managed by different ECMO approaches [16-18].

The aim of our experimental study was to determine how femoro-femoral (FF) compares to femoro-subclavian (FS) VA ECMO in producing adequate carotid and coronary blood flow as well as cerebral and myocardial oxygenation in a pig model of prolonged cardiac arrest, and whether the contribution of IABP in these ECMO approaches is significant. We further investigated whether both FF and FS ECMO assure adequate coronary perfusion pressure (CoPP) and offer a reasonable resuscitability despite a prolonged period of ventricular fibrillation (VF).

We hypothesized that FF ECMO might better assure carotid blood flow than FS ECMO and FS ECMO being more proximal will better supply coronary arteries. We also anticipated that IABP will improve both carotid and coronary blood flows. We further hypothesized that VA ECMO will provide sufficient myocardial oxygen support and CoPP reflected in a reasonable resuscitability.

## Materials and methods

This study was approved by the First Faculty of Medicine Institutional Animal Care and Use Committee and performed at the Animal Laboratory, Department of Physiology, First Faculty of Medicine, Charles University in Prague in accordance with Act No 246/1992 Coll., on the protection of animals against cruelty.

Twelve crossbred (Landrace × large white) female pigs (*Sus scrofa domestica*), four to five months old, mean body weight 50.3 ± 3.4 kg, were used in the study. After 24 h of fasting, anesthesia was induced by azaperone (2 mg/kg IM) followed by atropine sulphate (0.02 mg/kg IM) and ketamine hydrochloride (15 to 20 mg/kg IM). Anesthesia was continued with initial propofol and morphine boluses, (2 mg/kg IV and 0.1 to 0.2 mg/kg IV, respectively) and animals were orotracheally intubated. Continuous IV infusion of propofol (8 to 10 mg/kg/h) combined with morphine (0.1 to 0.2 mg/kg/h) IV were used to maintain anesthesia, the depth of which was regularly assessed by photoreaction and corneal reflex. Initial rapid IV infusion of 1,000 mL of normal saline was given intravenously, followed by continuous IV drip of 200 to 500 mL/h to reach and maintain central venous pressure of 3 to 7 mmHg. Whenever needed, mainly during initial launching of ECMO, additional crystalloids were administered as rapid IV boluses of 100 to 250 ml of normal saline. Unfractionated heparin (100 U/kg IV) was given as a bolus after sheaths placement followed by 40 to 50 U/kg/h continuous IV drip to maintain activated clotting time of 180 to 250 s (values were checked every hour with Hemochron Junior+, International Technidyne Corporation, Edison, NJ, USA).

For methods on ventilation, hemodynamic monitoring, high-frequency burst method to induce VF, laboratory values determination, cardiac O_2 _extraction determination, IABP institution and ECMO console, and circuit and cannulation, please refer to Additional file 1.

Brain and peripheral regional oxygen saturation levels were measured by near infrared spectroscopy (NIRS) using an INVOS Cerebral/Somatic Oximeter (Covidien, Boulder, CO, USA). Two spectroscopy sensors were used, one overlying the forehead and the other attached to the calf, opposite to the side where an arterial femoral ECMO cannula was inserted.

Coronary and carotid direct blood flow velocity measurement (see Additional file 2) was performed with a Doppler flow wire using ComboMap Pressure and Flow Measurement System (Volcano Corporation, Rancho Cordova, CA, USA). Doppler flow wires were inserted into straight proximal segments of coronary and carotid arteries through the guiding catheters for percutaneous coronary or carotid interventions, 2 to 3 cm behind the catheter orifices. A blood flow Doppler signal was obtained and analyzed in real-time, blood flow velocity was measured in cm/sec as an average peak value (APV) obtained from five consecutive instantaneous peak velocity (IPV) measurements. A mean APV during last minute of respective five-minute sampling periods were used for averaging. Values were stored for further off-line analysis and APV considered as a surrogate marker of coronary artery blood flow [19,20].

### Experimental protocol

Under fluoroscopic guidance, a coronary 6 F AR1 guiding catheter (Cordis, Miami, Fl, USA) was placed into the ostium of the left main coronary artery and a 6 F Headhunter carotid access or RCB guiding catheter (Cordis) was placed directly into the left carotid artery, or through the bicarotid trunk whenever present, 1 to 2 cm behind the ostium. An IABP balloon was inserted into the descending aorta and Swan-Ganz and coronary sinus catheters were advanced under fluoroscopic control.

**Figure 1:**
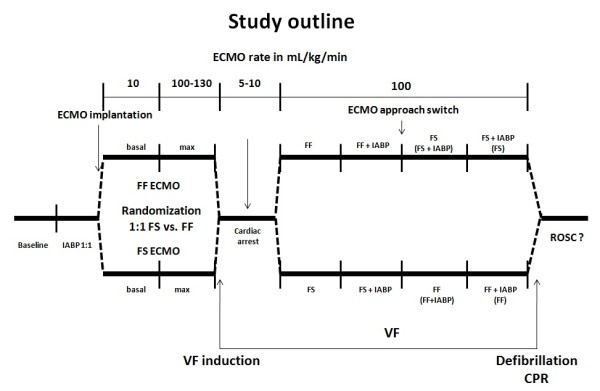
**Outline of the study protocol**. For an explanation see text. Vertical bars represent 15-minute intervals for respective measurement periods. CPR, cardiopulmonary resuscitation; ECMO, extracorporeal membrane oxygenation; FF, femoro-femoral; FS, femoro-subclavian; IABP, intraaortic balloon countepulsation; VF, ventricular fibrillation; ROSC, return of spontaneous circulation.

The right subclavian artery was surgically exposed and a 15 F ECMO cannula was inserted with its tip positioned to the origin of the right subclavian artery for later extracorporeal circuit connection. The cannula was clamped and continuously flushed with heparinized saline to avoid thrombosis. Thereafter, Doppler flow wires were inserted through guiding catheters to be positioned approximatelly 3 to 5 cm above the origin of the left carotid artery and in the proximal straight segment of the circumflex coronary artery, see Additional file 2. The Doppler wires were carefully manipulated and placed for obtaining the best possible ultrasound signals. The protocol consisted of 15-minute intervention intervals outlined in Figure 1. Following stabilization, all hemodynamic and flow values in each of these intervals were obtained every five minutes and averaged, the blood was always drawn at the end of each interval. The baseline interval was followed by the IABP set to a 1:1 mode to adjust for the best possible augmentation. Thereafter, the ECMO cannulae were inserted according to randomization: in the FF arm, both venous and arterial femoral cannulae; in the case of the FS arm, only a venous femoral cannula was inserted and the previously prepared subclavian cannula was used. Fluoroscopically, femoral artery ECMO cannula position was controlled not to be in close proximity to the IABP balloon to avoid interference with aortic ECMO flow. Initially, a basal mode of < 10 mL/kg/minute blood flow was set for stabilization. Next, a maximal ECMO flow mode was defined by a blood flow rate of 100 to 130 mL/kg/minute. Then, cardiac arrest was commenced by VF induction using programmed ventricular stimulation and ECMO flow was set to 5 to 10 mL/kg/minute. After 15 minutes of cardiac arrest, the ECMO flow was increased to reach the target flow of 100 mL/kg/minute as soon as possible. After finishing the ECMO interval and all data retrieval, IABP was switched on to an internal mode of 100 inflations/minute. Then, an ECMO switch procedure was carried out and all values were again retrieved from ECMO and ECMO + IABP configurations. Vasopressors were not used in these ECMO-treated phases of cardiac arrest. After all values and blood specimens were obtained, standard CPR including chest compressions, whenever needed, were started with 270 J biphasic DC defibrillation (TEC-550, Nihon Kohden, Japan). Return of spontaneous circulation (ROSC) was evaluated based on the following definition: a supraventricular rhythm with hemodynamically effective pulsations regardless of ECMO flow with mean arterial pressure of 60 mmHg. The evaluation took place at 5 and 60 minutes following first defibrillation. An ECMO weaning trial was performed regularly and until 60 minutes post CPR, hemodynamic tolerance was tested. Hypotension was eventually corrected with standard norepinephrine dosing administered continuously by an IV drip. Thereafter, the animal was euthanized by morphine and propofol overdose followed by intravenous potasium chloride 1 mmol/kg.

### Statistical analysis

Descriptive data are presented as median (interquartile range) of measured values or as median (interquartile range) of individual baseline percentages of each experiment. The median of baseline percentage is not percent expression of absolute value median. It is a median of baseline percentage from each experiment. Repeated analysis of variance measures were used to assess the between-subject effect within each arm of the study (that is, we assessed the effect of the ordering of ECMO procedures as one arm started with FF ECMO, whereas the other started with FS ECMO; Figure 1). No difference was noticed. We then pooled the corresponding methods from both arms to assess the differences. Each animal was used as its own control, and paired comparisons were made between respective approaches. A Friedman test and Tukey's *post hoc *analysis were performed for different analyses of ECMO approaches. *P*-values < 0.05 were considered statistically significant. All statistical analyses were performed by MedCalc software version 11.6. (MedCalc Software, Broekstraat 52, 9030 Mariakerke, Belgium) and MATLAB Statistics Toolbox (MathWorks Inc., Natick, MA, USA).

## Results

Out of 12 animals entered in the study, 11 animals successfully completed the full protocol and all data were retrieved and used for evaluation. The single animal that did not complete the study developed massive retroperitoneal bleeding during venous ECMO cannula insertion and died of refractory hemorrhagic shock. The initial preparation period encompassing all cannulations and surgical implantation of the subclavian ECMO cannula, ECMO tolerance and effectivity testing lasted 5:01 hours (4:28, 5:33), cardiac arrest 15:33 min (13:59,16:08) and ECMO switch procedure 2:12 minutes (0:29, 3:15). Overall protocol duration was 7:18 hours (6:30, 7:55). Average fluid (saline) loading during the whole experiment was 4.622 ± 1.103 ml to reach target central venous pressure values and to avoid ECMO underfilling. In all animals, target ECMO flow of 100 mL/kg/minute has been reached, in three animals (#1, 6, 11) pulmonary edema developed.

### Carotid and coronary blood flow velocities

**Figure 2:**
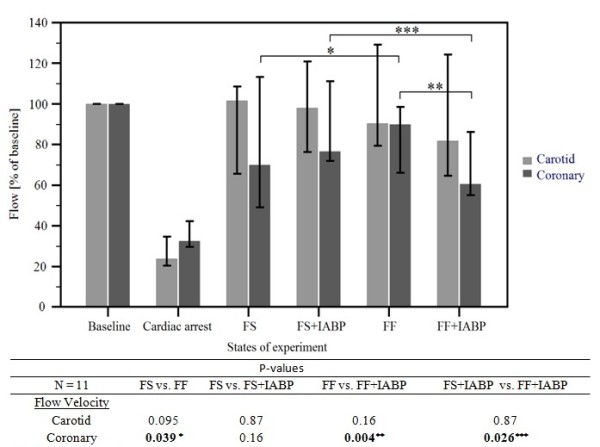
**Carotid and coronary blood flow velocities relative to baseline**. Values are expressed as median percentages of baseline with 25- and 75-percentiles represented by the vertical bars (for actual values see Table 1) along with *P*-values for respective comparisons at the bottom. Statistically significant differences in bold. Cardiac arrest values are significantly different to all other values, comparisons not shown.

Carotid and coronary blood flow velocities presented as average peak velocities are enumerated in Table 1. Absolute values give velocities at baseline, cardiac arrest and different ECMO regimens. Cardiac arrest values of 12 (7.0, 12.0) cm/sec for carotid and 8 (7.0, 9.8) cm/sec for coronary flow velocities reflect very low flows generated by basal ECMO setting to prevent thrombosis within the ECMO circuit and simulating a "low-flow" cardiac arrest state. Relative carotid and coronary blood flow velocities according to different ECMO regimens during the continued cardiac arrest treated by ECMO are graphically outlined with respective statistical evaluation in Figure 2.

**Table 1:** Carotid and coronary blood flow average peak velocities during different ECMO regimens.

N = 11	Baseline	Cardiac arrest	FS	FS+IABP	FF	FF+IABP
**Flow Velocity **(cm/s)						
Carotid	36.7 (33.3, 46.8)	12.0 (7.0, 12.0)	32.0 (25.5, 43.5)	32.3 (27.3, 39.0)	35.0 (25.7, 48.0)	27.3 (24.7, 37.3)
Coronary	21.7 (19.3, 24.4)	8.0 (7.0, 9.8)	14.0 (10.3, 25.0)	17.0 (15.9, 23.0)	18.7 (16.4, 21.8)	15.7 (11.5, 18.8)
**Flow Velocity** (% of baseline)						
Carotid	---	23.8 (20.5, 34.6)	101.5 (65.6, 108.6)	98.0 (76.4, 120.9)	90.3 (79.4, 129.1)	81.8 (64.7, 124.3)
Coronary	---	32.6 (29.6, 42.3)	70.0 (49.1, 113.2)	76.7 (71.9, 111.2)	90.0 (66.1, 98.6)	60.7 (55.1, 86.2)

**Assessment performed by Doppler flow wire measurements in cm/s (upper part) and as a percentage of baseline (bottom part)**. FS, femoro-subclavian; FF, femoro-femoral; IABP, intraaortic balloon counterpulsation; The median of baseline percentage is not percent expression of absolute value median. It is a median of baseline percentage from each experiment.

Carotid flow velocities in all ECMO approaches regardless of IABP presence reached values higher than 80% of baseline flow and were near to 100% of baseline values in both FS and FS + IABP configurations. Addition of IABP to FF ECMO did not change the carotid flow significantly.

Coronary flow velocities are lower, reaching about a half of the carotid velocities. Only the FF regimen assures values over 80% of baseline. Addition of IABP to the FF approach caused a statistically significant decrease in coronary flow velocity, *P *= 0.004. The FS ECMO approach reached only 70.0% (49.1, 113.2) of the baseline flow, which is significantly lower than 90.0% (66.1, 98.6) of FF ECMO, *P *= 0.039. Addition of IABP to FS did not significantly change the coronary blood flow velocity. Comparison of both IABP regimens shows a statistically significant difference, FS +IABP vs. FF + IABP 76.7% (71.9, 111.2) vs. 60.7% (55.1, 86.2), *P *= 0.026. Thus, the FF+IABP approach reached the lowest coronary flow velocities in this cardiac arrest setting.

### Brain and peripheral oxygen saturations

Brain and peripheral regional oxygen saturations are demonstrated in Table 2, and the values relative to baseline during different ECMO regimens and statistical evaluation are outlined in Additional file 3. Both brain and peripheral regional oxygen saturations dramatically decreased immediately after induction of VF cardiac arrest to 23.0% (15.0, 32.3), *P *= 0.001, and 34.0% (23.5, 34.0), *P *= 0.001, in absolute values versus baseline, respectively, and after restoration of flow rapidly improved. Thus, for brain saturations both ECMO regimens reached values exceeding 80% of baseline and similar ranges apply for IABP assistance. Comparably similar values were detected also for peripheral saturations. None of the comparisons for brain or peripheral saturations differed significantly.

**Table 2:** Measured peripheral and brain regional oxygen saturations in % during different ECMO regimens.

N = 11	Baseline	Cardiac arrest	FS	FS+IABP	FF	FF + IABP
**Oxygen saturation **(%)						
Peripheral	56.7 (49.2, 62.7)	34.0 (23.5, 34.0)	49.7 (48.4, 55.5)	48.7 (44.7, 52.5)	54.0 (40.2, 57.6)	46.7 (41.1, 56.2)
Brain	56.0 (50.5, 58.1)	23.0 (15.0, 32.3)	49.0 (42.8, 52.4)	52.0 (47.2, 53.2)	50.7 (41.9, 58.0)	49.0 (42.4, 55.6)
**Oxygen saturation** (% of baseline)						
Peripheral	---	53.7 (42.0, 63.5)	91.4 (79.4, 99.9)	86.9 (75.4, 96.1)	92.1 (76.4, 103.5)	94.5 (73.3, 98.2)
Brain	---	39.2 (30.3, 55.9)	86.4 (83.4, 91.7)	90.1 (83.6, 99.8)	93.8 (80.8, 102.1)	91.9 (82.2, 97.6)

**Absolute values outlined in upper part and percentage of baseline values in bottom part**. FS, femoro-subclavian; FF, femoro-femoral; IABP, intraaortic balloon counterpulsation; The median of baseline percentage is not percent expression of absolute value median. It is a median of baseline percentage from each experiment.

### Coronary perfusion pressure progress during the experiment

**Figure 3:**
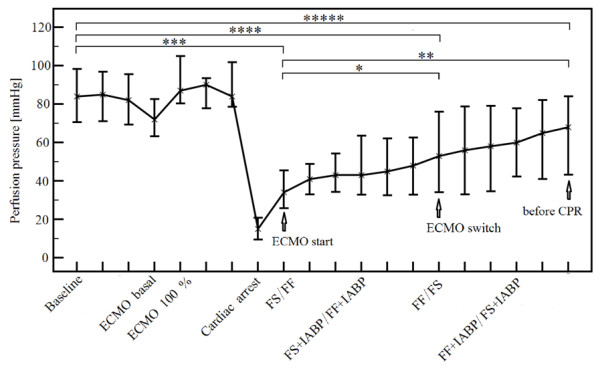
**Coronary perfusion pressure during the whole protocol**. Pooled from all eleven animals as medians with 25- and 75-percentiles represented by vertical bars. See the gradual rise of perfusion pressure after the ECMO start. Respective statistical comparisons delineated by horizontal bars, statistically significant differences in bold:*****P = 0.076;****P = 0.007; *** *P *< 0.001; **P = 0.003; *P = 0.037.

**Figure 4:**
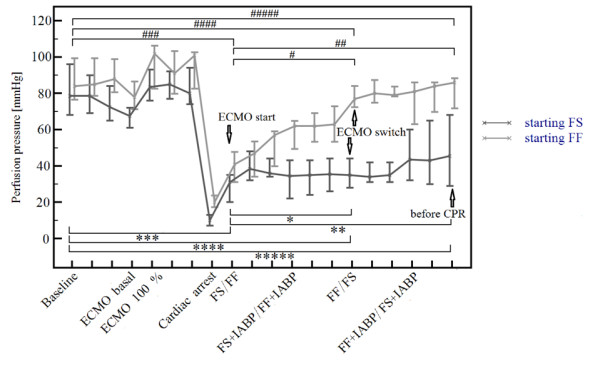
**Coronary perfusion pressure progression over time and differences in respective ECMO approaches according to randomization**. Values in bold mean statistically significant difference. The pressure differences **between **the respective arms on "ECMO start", "ECMO switch" and "before CPR" are 10, 42 and 40.5 mmHg, respectively, see Table 3 for absolute values (*P *= 0.193 for "ECMO start", ***P *= 0.045 **for "ECMO switch" and ***P *= 0.008 **for "before CPR" values). Statistical evaluation for CoPP progression **within **the respective arms is delineated by horizontal bars with statistical evaluation as follows. *P*-values for FF arm: ^#####^*P *= 0.7;^####^*P *= 0.39; **^### ^*P *= 0.002; ^##^*P *= 0.006; ^#^*P *= 0.02**. *P*-values for FS arm: *****P = 0.041, ****P = 0.004, ***P < 0.001, **P = 0.11, *P = 0.49.

Immediatelly after induction of VF and cardiac arrest, the CoPP decreased from a baseline of 84 mmHg (69.7, 97.0) to 15 mmHg (9.5, 20.8). Of note, animals randomized to FS starting cohort, tended to have nonsignificantly lower pre-arrest CoPP. The first CoPP value on ECMO was 34 mmHg (25.8, 45.5) and later during the protocol gradually rose. This phenomenon of a gradual rise with time was detected in almost all animals except for two of them, see Figure 3 and Additional file 4 and was present both in the FS and FF arms of the protocol. A more pronounced increase in CoPP values was detected in the FF ECMO arm (see Figure 4). Table 3 demonstrates the actual progress of CoPP, MAP and CVP values during the experiment and their respective differences according to a randomization assignment. Baseline pulmonary capillary wedge pressure and cardiac output values are also shown to demonstrate comparable baseline values in respect to treatment arms. Statistical evaluations comparing baseline CoPP values with values reached before CPR and ECMO onset values with those before CPR values are outlined in Figures 3 and 4. The final CoPP value at the end of the protocol, that is, after ECMO-treated cardiac arrest of more than two hours on average, reached almost the baseline value. Animals started on FF ECMO completed the protocol with identical CoPP values as at the baseline, with a steep rise in CoPP during the FF ECMO part of the protocol. The difference of initial CoPP ECMO values between the FF versus FS treatment arms was 10 mmHg (41 minus 31), *P *= 0.193, increased to a significant difference of 42.0 mmHg (77.0 minus 35.0) at the time of ECMO switch, *P *= 0.045, and remained significantly different before CPR, that is, 40.5 mmHg (86.0 minus 45.5 mmHg), *P *= 0.008. Animals starting on FS ECMO had a similar rise in CoPP during the later phase of the protocol, after a switch to FF ECMO. For more statistical differences, please see Figure 4.

**Table 3:** Coronary perfusion pressure, mean arterial pressure and central venous pressure progress during the experiment.

CoPP (mmHg)	Baseline	Cardiac arrest	ECMO start	ECMO switch	before CPR
Both Arms Together (N = 11)	84.0 (69.7, 97.0)	15.0 (9.5, 20.8)	34.0 (25.8, 45.5)	53.0 (34.0, 76.0)	68.0 (43.3, 84.0)
FS starting (N = 6)	78.5 (68.5, 89.3)	10.0 (7.5, 12.5)	31.0 (20.0, 35.0)	35.0 (28.0, 44.0)	45.5 (29.0, 68.0)
FF starting (N = 5)	85.0 (80.0, 99.0)	20.0 (18.0, 21.0)	41.0 (31.0, 47.8)	77.0 (72.3, 84.0)	86.0 (71.8, 88.3)

**MAP (mmHg)**					

Both Arms Together (N = 11)	88.0 (75.8, 102.0)	24.0 (19.3, 32.5)	41.0 (35.3, 51.0)	61.0 (40.5, 83.5)	75.0 (50.0, 88.3)
FS starting (N = 6)	81.2 (72.0, 96.0)	20.5 (17.0, 23.0)	40.0 (34.0, 51.0)	41.0 (35.0, 51.0)	52.0 (36.0, 75.0)
FF starting (N = 5)	89.7 (83.6, 104.5)	31.0 (28.5, 35.8)	49.0 (38.5, 52.5)	85.0 (78.5, 88.0)	90.0 (77.3, 95.3)

**CVP (mmHg)**					

Both Arms Together (N = 11)	5.2 (4.4, 6.0)	11.5 (10.0, 12.0)	7.0 (5.3, 8.0)	7.0 (6.3, 7.8)	7.0 (6.3, 7.0)
FS starting (N = 6)	5.3 (4.0, 6.0)	10.0 (9.8, 12.0)	6.5 (5.0, 9.0)	7.0 (7.0, 8.0)	7.0 (7.0, 8.0)
FF starting (N = 5)	4.7 (4.5, 6.4)	12.0 (10.5, 12.0)	7.0 (5.5, 8.0)	7.0 (5.3, 7.3)	7.0 (4.8, 7.0)

**PCWP (mmHg)**					

Both Arms Together (N = 11)	9.0 (8.0, 10.5)				
FS starting (N = 6)	9.0 (8.0, 10.0)				
FF starting (N = 5)	9.0 (7.5, 11.3)				

**CO (L/min)**					

Both Arms Together (N = 11)	6.4 (5.4, 7.5)				
FS starting (N = 6)	6.2 (5.4, 7.5)				
FF starting (N = 5)	6.8 (5.5, 7.5)				

Baseline pulmonary capillary wedge pressure and cardiac output values are also shown in the lower part. None of the baseline differences between treatment arms are statistically significant.

FS, femoro-subclavian; FF, femoro-femoral; CO, cardiac output; CoPP, coronary perfusion pressure; CPR, cardiopulmonary resuscitation; CVP, central venous pressure; MAP, mean arterial pressure

### Lactate levels and myocardial oxygen extraction

**Figure 5:**
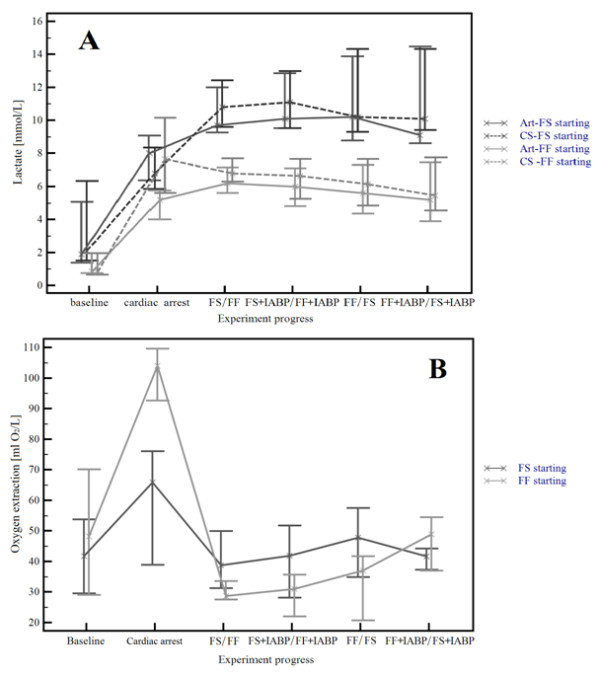
**Coronary artery and coronary sinus lactate levels and myocardial oxygen extraction during the protocol**. Lactate levels (panel A) steeply rise during cardiac arrest in both arms. In the FS arm, lactate rise persists even during the first ECMO treatment and reaches significantly higher plateau levels than in the FF arm both for arterial and CS values (*P *= 0.016 and *P *= 0.035, respectively). Significantly higher levels in the FS arm persist during the rest of the protocol, however in both arms a downward trend can be detected, though more pronounced in the FF arm. Myocardial oxygen extraction in ml O_2_/L (panel B) markedly increases during cardiac arrest followed by a steep decline after starting both ECMO regimens. Values after ECMO start are not significantly different (see text).

Lactate levels measured in the coronary artery ostium and in the coronary sinus are outlined in the upper part of Figure 5. Interestingly, we have detected significantly higher levels of lactate in animals started on FS ECMO following cardiac arrest in all post arrest periods (*P *= 0.016 and *P *= 0.035 for differences in arterial and coronary sinus lactate levels, respectively). Moreover, after a steep increase of lactate levels during cardiac arrest and the first ECMO period, a plateau level and then a slight decrease could be detected, which was more prominent for the FF ECMO approach. The lower part of Figure 5 demonstrates O_2 _extractions outlined similarly as lactate levels. After a steep increase in O_2 _extraction during cardiac arrest a very deep decline of O_2 _extraction related to ECMO initiation is apparent and these low extractions remain further with no statistically significant differences between the respective ECMO arms (*P *for difference = 0.547).

### Resuscitability

Resuscitability of the animals was high. Out of 11 animals, we gained 5 minutes ROSC in 8 animals (73%). 60 minutes ROSC was achieved in 8 (73%) animals (Additional file 5); however, see animal #3, in whom initial hypotension (5 minutes ROSC assessment with mean arterial pressure of 58 mmHg) improved markedly to fullfil criteria for 60-minute ROSC. However, the ECMO support of > 50 mL/kg/min was prolonged to keep perfusion pressure in 5 of 11 animals (#3, 4, 6, 8, 11). In the rest of them, ECMO was weaned quickly after initial CPR. Two animals could not be resuscitated to reach ROSC, one developed pulseless electrical activity after the first defibrillation (animal #1), the other suffered refractory VF despite six defibrillations (animal #2). Interestingly, these animals with unsuccessful CPR had steep decreases in coronary perfusion pressure during the ECMO phases, as described above, see Additional file 4.

## Discussion

Using a pig model replicating a VA ECMO treated cardiac arrest due to prolonged VF, we confirmed that this approach used in some specialized centers for urgent organ support (via percutanous FF insertion) sufficiently assures both cerebral and myocardial perfusion, oxygenation, and rapidly improves the post arrest metabolic state. In contrast to our hypothesis, a FS approach (despite sufficiently maintaining brain perfusion and oxygenation) was not an optimal option for maintaining coronary perfusion. Moreover, this latter approach offered worse myocardial metabolic recovery. We also tested an often encountered clinical combination of IABP with VA ECMO. We found that when used along with the FF ECMO approach, IABP significantly impaired coronary perfusion in comparison to FF ECMO alone. Furthermore, we have shown that in the FS ECMO approach, coronary flow after addition of IABP remained low, not reaching even 80% of the baseline level.

This is an important finding, because the cause of arrest is of cardiac origin in a majority of cases [3,12]. It is well described that besides the primary cardiac disease, nonpulsatile ECMO flow may adversely affect cardiac performance in critical states, including hypoxemic blood perfusion of the coronary circulation due to a pulmonary dysfunction [21], changes in load-dependent contractile function [22] and pure mechanical negative effect on LV function [23]. A controversial effect on afterload changes has also been described [24,25]. It appears that cannulation selection, that is, subclavian/carotid arterial ECMO cannula position and the magnitude of bypass flow also play a critical role in coronary blood flow, depending on the distance from the coronary arteries, and thus, "availability" of the oxygenated blood [26-28]. Smith *et al. *[26] have shown in young lambs, by using labeled albumin microspheres injected into carotid perfusion cannula, that relative coronary blood flow was significantly less than in the control group. Kato *et al. *[27] demonstrated in puppies, that coronary arterial flow in VA ECMO with carotid arterial cannula decreased even proportionally to ECMO flow increase despite no significant changes in the mean or diastolic pressures in the ascending aorta. And most interestingly, Kamimura *et al. *[28], in neonatal dogs, proved the difference in coronary blood flow distributed from the proximal arterial cannula (1 cm above aortic valve) being significantly higher than that from the distal cannula (ostium of the brachiocephalic trunk). He concludes that proximal arterial cannula appears necessary to provide sufficient oxygenated blood to the coronary circulation during V-A ECMO. However, animals in these three experimental protocols were not in cardiac arrest. We speculate that not only distance, but also the angle under which the outflow arterial cannula in FS approach is located, may play the role, causing prograde flow in aortic arch, thus underperfusing coronary arteries. These considerations are of critical importance in cardiogenic shock with persistent lung failure treated by peripheral ECMO to avoid coronary perfusion by poorly oxygenated blood ejected from the left ventricle.

It has also been demonstrated that added pulsatility to continuous flow mechanical support improves organ perfusion in terms of blood flow, flow velocity in coronary artery [29,30], energy equivalent pressure and surplus hemodynamic energy, though not having influenced mean carotid pressure [31,32], and even improves a renal perfusion [33]. However, in most of these experiments, a central cannulation approach was used for both inflow and outflow cannulae. This surgical approach is usually not used in cardiac arrest patients [17,18]. We, therefore, investigated whether pulsatility represented by IABP might play a similar beneficial role also in peripheral configuration of FF vs. FS ECMO in the cardiac arrest model. Our results show that in this situation, IABP may have an unfavorable effect on macrocirculation in the FF configuration. We speculate that an intermittent aortic occlusion by the IABP balloon may diminish blood flow available for the aortic root (and thus for coronary arteries), more than for the aortic arch (and thus for the carotids). These results are in accordance with a report by Sauren [34], where addition of IABP in severely hypotensive ECMO-treated animals was not beneficial; however, the animals were not in cardiac arrest. On the other hand, the subclavian approach seems to be suitable for IABP assistance because the outflow cannula is located proximal to the IABP balloon. Drakos *et al. *[35] showed a similarly beneficial effect of IABP pulsatility on coronary artery flow in a proximally implanted nonpulsatile centrifugal pump in an open chest pig model of profound cardiogenic shock. Accordingly, the direction of outflow arterial flow and distance from coronaries is also of importance [28]. Our results add to the controversy about the effect of IABP-generated pulsatility both on macro- and microcirculation; in small non-randomized clinical studies, Jung [36,37] and den Uil [38] have reported on a favorable effect of IABP on microcirculation in cardiogenic shock, but Mustermann [39] showed paradoxically improved microvascular flow after withdrawing the IABP. Microcirculation changes definitely play a key role in critical states [40-42] and the role of pulsatility and different support combinations (that is, central vs. peripheral, subclavian vs. femoral) on microcirculatory changes remains to be further studied.

An important observation of our study is that VA ECMO significantly increases coronary perfusion pressure over time, mainly in the FF configuration. CoPP actually reached the baseline values by the end of our protocol, that is, approximately two hours after the ECMO onset (Figures 3 and 4, Additional file 4 and Table 3). When pooling data from all animals, the CoPP increased progressively from 15.0 mmHg at the end of the cardiac arrest to 34.0 mmHg five minutes after starting ECMO, rose to 53.0 mmHg at the ECMO switch and continued to rise to 68.0 mmHg before CPR, not different from baseline. The increases in CoPP were more pronounced during the FF ECMO period of the protocol, which was responsible more than FS ECMO for an overall CoPP increase. In contrast to FF ECMO, where baseline to before CPR values reached the same values, in the FS ECMO starting cohort, the CoPP at the end of the protocol still remained significantly lower compared to baseline (45.5 vs. 78.5 mmHg, respectively, *P *= 0.041). To our knowledge, this phenomenon of the CoPP increase over time during VA ECMO treated cardiac arrest has not been described before. This finding is important, because CoPP is a key prerequisite for ROSC in prolonged cardiac arrest [43,44]. We can only speculate about the pathophysiological mechanism behind the low CoPP during ECMO start and gradual CoPP increase with time on ECMO; however, vasoplegia with low peripheral resistance post low flow cardiac arrest followed by improved vasoreactivity during further ECMO reperfusion offers a reasonable explanation. We intentionally did not use vasopressors in this phase of the experiment in order to observe the „natural" course of ECMO reperfusion. In human care, we are used to keeping the perfusion pressure (that is, MAP) within optimal range with norepinephrine to assure adequately both cerebral flow and perfusion pressure.

The adequacy of CoPP was reflected by a high resuscitability of our animals (see Additional file 5), despite a rather strenuous and prolonged protocol. Of note, we used a demanding definition for ROSC, as stated earlier. This definition is more strict and difficult to accomplish in comparison with other ROSC definitions used in similar settings, that is, "stable pulse with a systolic arterial pressure > 60 mmHg" [45] or "systolic pressure ≥ 80 mmHg for at least one minute continuously at any time during the resuscitation effort" [44]. Thus, we consider the VA ECMO-mediated CoPP increase to be responsible for an excellent resuscitability and a considerable hemodynamic stability after early ROSC. These findings are in accordance with myocardial metabolism and recovery as outlined in Figure 5. After a steep increase during cardiac arrest and the initial phase of ECMO treatments, lactate levels corresponding to global post arrest ischemia reach a peak value followed by a plateau level and then a gradual decline. Interestingly, the peak and plateau lactate levels are significantly lower in the FF ECMO arm, possibly reflecting better organ perfusion. Lactate data are in accordance with myocardial O_2 _extraction: a steep increase during untreated cardiac arrest reflecting insufficient coronary perfusion is immediatelly reversed after initiating ECMO. Interestingly, no significant difference was noted in O_2 _extraction related to ECMO treatment arms. The possible explanation might be that despite detected lower coronary blood flow and perfusion pressure in the FS ECMO arm, low O_2 _extraction, most probably, reflects a sustained satisfactory O_2 _supply. This assumption might be supported by the threshold character of both myocardial O_2 _balance and CoPP, meaning that critically low levels have not been reached. However, this observation has to be interpreted with caution, because the difference in O_2 _extraction has been detected to be significant between treatment arms already during the cardiac arrest period despite similar baseline values. In the FS ECMO arm, still sufficient CoPP has been generated during the whole protocol, see Table 3. Such a value is well above the CoPP considered to be accompanied by a high probability of ROSC [43,44].

We also designed this experimental study with the intention to evaluate cerebral and coronary reperfusion in terms of appropriate reoxygenation. As shown in Additional file 3 and Table 2, both FF and FS ECMO regimens offered adequate (that is, not unintentionally high) brain and peripheral oxygenations expressed by regional O_2 _saturations immediatelly after ECMO initiation. Re-oxygenation on ECMO can be easily controlled when using on-line monitoring of blood gases as in our protocol, thus avoiding the possible adverse effects of arterial hyperoxemia on outcome after reperfusion/ROSC [46,47].

### Study limitations

ECMO and IABP have been implanted prior to induction of cardiac arrest, which is not the „real" clinical scenario model. However, urgent ECMO implantation (especially the subclavian approach) during cardiac arrest is a complex invasive procedure that would probably interfere with the primary aim of this study, that is, proper and accurate determination of coronary and carotid blood flow velocities following the cardiac arrest period. Blood flow measurements were not obtained directly using a flow probe, which, however, would need an open chest model. Instead, we assumed that Doppler wire acquired APV offers a good linear relation to blood flow in proximal LCx segments [19] and thus can be used as a surrogate marker of blood flow, mainly for detection of flow velocity changes assuming nonsignificant changes in a vessel diameter [20]. We controlled for potential confounding factors influencing vessel diameter like pCO_2_, intravascular volume, level of vasocontriction and ECMO flow. Obtained APV values are also potentially influenced by proper wire positioning. To overcome this limitation, we insisted on a very careful wire handling to minimize position changes during the course of the study protocol.

In our cardiac arrest period, the "no-flow" phase was substituted entirely by a "low-flow" phase due to ECMO basal flow. However, basal flow was very low (targeted to 5 to 7, never over 10 mL/kg/minute) and monitored parameters (regional cerebral saturation, lactate, O_2 _extraction) demonstrated profound changes corresponding to severe organ hypoperfusion. From the technical reasons, we also had to omit the mechanical chest compressions during the initial „low-flow" cardiac arrest, because Doppler wire technology is extremely sensitive to movements and any even very delicate changes in position, that is, mechanical compressions, would result in losing the proper position to catch and exactly record the post arrest flows.

For brain oxygenation, we used NIRS technology to monitor regional cerebral saturation with its inherent limitations; that is, not a global cerebral oxygenation assessment, not a deep tissue penetration, only relative values in one subject and their change in time can be used for evaluation. However, this is a very easy, noninvasive tool for detection of even minor changes in regional brain saturation and its use has been validated both in a pig CPR model [48] and for prognostic assessment in OHCA survivors on admission to hospital [49].

Different arterial cannula size used for FS (15 F) and FF (17 F) approach because of the vessel size is another limitation; however, target ECMO flow of 100 mL/kg/minute was achieved and did not differ in both approaches. And finally, our biomodel was represented by a breed that has been validated for simulation of human cardiac arrest and resuscitation [48,50]. Nonetheless, pig anatomy differs from human and the presence of bicarotid trunk instead of two separate carotid arteries and the angle under which subclavian artery leaves the aorta might possibly influence our results.

## Conclusion

In a simulated model of VA ECMO-treated prolonged cardiac arrest, our experimental study showed several important findings. First, the study confirmed that the increasingly used first line approach for urgent organ support in refractory arrest (that is, FF ECMO) sufficiently assures both brain and myocardial perfusion, oxygenation and rapidly improves the post-arrest metabolic state. Second, the FS approach, while sufficiently maintaining brain perfusion, may not be an optimal option to maintain coronary perfusion and offers worse myocardial metabolic recovery. Third, and most importantly, the addition of IABP to an established VA ECMO is a controversial intervention: in the FF ECMO approach, it significantly impairs coronary perfusion and in the FS approach, coronary perfusion remains the same, still not reaching 80% of the pre-arrest value. Fourth, VA ECMO significantly increases coronary perfusion pressure with time. Of interest, in animals starting with FF approach pre-arrest values of coronary perfusion pressure have been reached at the end of our protocol. However, a trend towards increasing coronary perfusion pressure with time is apparent also in FS ECMO. The VA ECMO-mediated CoPP increase is probably responsible for excellent resuscitability and considerable hemodynamic stability after early ROSC. Thus, generally, the VA ECMO-based approach to refractory cardiac arrest provides a time span for interventions to correct the cause of the arrest and assures both sufficient vital organ perfusion and sustained resuscitability.

## Key messages

• In a pig model of prolonged cardiac arrest, a femoro-femoral venoarterial ECMO-based approach provides adequate cerebral and myocardial perfusion and oxygenation.

• The combination of IABP with FF ECMO may impair coronary perfusion.

• Both FF and FS ECMO assure adequate coronary perfusion pressure and sustained resuscitability. Moreover, FF ECMO provides CoPP rise with time and provides rapid myocardial metabolic recovery.

## Abbreviations

APV: average peak value; CA: cardiac arrest; CaBF: carotid blood flow; CoBF: coronary blood flow; CoPP: coronary perfusion pressure; CPR: cardiopulmonary resuscitation; CVP: central venous pressure; DC: direct current; ECMO: extracorporeal membrane oxygenation; E-CPR: extracorporeal cardiopulmonary resuscitation; FF: femoro-femoral; FS: femoro-subclavian; IABP: intraaortic balloon counterpulsation; IM: intramuscular; IV: intravenous; LCx: left circumflex artery; MAP: mean arterial pressure; Min: minute; NIRS: near infrared spectroscopy; OHCA: out of hospital cardiac arrest; PCWP: pulmonary capillary wedge pressure; PEA: pulseless electrical activity; ROSC: return of spontaneous circulation; SV: supraventricular; VA: venoarterial; VF: ventricular fibrillation.

## Competing interests

All authors have no commercial associations that might impose a conflict of interest.

## Authors' contributions

JB conceived the study, designed the study protocol, performed the main procedures during the study, and prepared and finalized the manuscript. MM, TS, SH and TK assisted during the study performance and drafted the article. MH and TB performed data acquisition and statistical analysis. MB, AL, PO and OK participated in a design of the study, contributed to the interpretation of the results, and helped to draft the manuscript. FM was responsible for ECMO institution. MA, AL and VM obtained research funding. All authors read and approved the final manuscript for publication.

## Supplementary Material

Additional file 1 **Methods**. Click here for file

Additional file 2 **Typical Doppler flow wire positions in carotid (panel A) and coronary (panel B) arteries**. Panel A shows subclavian 15 French ECMO cannula (long black arrow), carotid angiography performed through carotid guiding catheter (short black arrow) positioned in the ostium of left carotid artery (long white arrow) and the tip of the Doppler flow wire (short white arrow), also shown in the box detail without contrast agent injection. Panel B shows coronary angiography with left anterior descending (LAD) (small black arrow) and left circumflex arteries (LCx) (long black arrow) and similarly in detailed box a tip of the Doppler wire in the proximal straight part of LCx (black arrowhead). At the bottom shown typical Doppler wire tracings obtained during cardiac arrest and running VA ECMO from carotid (panel C, APV = 41 cm/sec) and coronary (panel D, APV = 22 cm/sec) arteries. APV = average peak value. Click here for file

Additional file 3 **Regional oxygen saturations measured by near infrared spectroscopy shown as percentage of baseline**. After a dramatic decrease during cardiac arrest, sufficient restoration of both brain and peripheral saturations by all treatments is demonstrated. No significant difference for any of the comparisons noted. For actual values see Table 2. FS, femoro-subclavian; FF, femoro-femoral; IABP, intraaortic balloon counterpulsation. Click here for file

Additional file 4 **Coronary perfusion pressure as measured in individual animals**. The two animals with pressure drop (#1 and #2) marked by black arrows. Click here for file

Additional file 5 **Resuscitability of animals, number of defibrillations and ROSC presence (YES/NO) at 5 and 60 minutes**. As vasopressors, epinephrine (Epi) and norepinephrine (Norepi) boluses followed by continuous IV drip were used only after defibrillations and CPR, doses given in mg for boluses and μg/kg/min for IV drip (norepinephrine). Click here for file
