# Supplementary material for: Coronary versus carotid blood flow and coronary perfusion pressure in a pig model of prolonged cardiac arrest treated by different modes of venoarterial ECMO and intraaortic balloon counterpulsation
Source: Crit Care. 2012 Mar 16;16(2):R50. doi: 10.1186/cc11254 (PMC3964801; doi:10.1186/cc11254)
Supplement: Additional file 1 — Methods. [file cc11254-S1.DOC]

**Ventilation** was provided by a Hamilton G5 ventilator (HAMILTON MEDICAL AG, Bonaduz, Switzerland) set to INTELLiVENT®-Adaptive Support Ventilation (ASV) mode. INTELLiVENT-ASV was set to maintain SpO_2_ of 95-99% and EtCO_2_ of 4.5-5.6 kPa. During the non-treated phase of cardiac arrest, the animals were not ventilated. In an ECMO treated phase of cardiac arrest, animals were ventilated with tidal volumes of 8-10 mL/kg, positive end-expiratory pressure 0.5 kPa and fraction of inspired oxygen 21%. Respiratory rate was adjusted to keep EtCO_2_ within above limits.

**Hemodynamic monitoring** was performed by using routine invasive pressure transducers (Truwave, Edwards Lifesciences, LLC, Irvine, CA, USA) to measure central venous pressure and pulmonary capillary occlusion pressure via inserted central venous line (internal jugular vein), Swan-Ganz catheter (via femoral vein) and aortic pressure via sheats, guiding catheters or a counterpulsation baloon tip. CoPP was determined as a difference between mean arterial pressure and central venous pressure. For monitoring the progress of coronary perfusion pressure during the experiment, the ECMO start values represent average values obtained 5 minutes after the start of each respective ECMO regimen, while the „before CPR“ values represent the last CoPP values immediatelly before VF defibrillation and CPR.

**A high-frequency burst method to induce VF** was performed with a diagnostic decapolar 6-Fr non-steerable catheter with 2-8-2 mm spacing (Response CSL, St. Jude Medical, St. Paul, MN, USA) advanced to the right ventricular apex through a femoral or jugular venous approach. The stimulation current amplitude was extended twice above a pacing threshold.

**Laboratory values** were determined by using point of care ABL 90 Flex Analyzer (Radiometer Medical, Åkandevej 21, DK-2700, Brønshøj, Denmark). Arterial blood samples were drawn from a coronary artery guiding catheter; its position was checked by angiography. Coronary sinus blood samples were drawn through an 8F sheath (Fast-cath SR0, St. Jude Medical, St. Paul, MN, USA) advanced to the coronary sinus under fluoroscopic guidance. The correct position of the sheath was verified by a typical coronary sinus signal pattern obtained with a diagnostic decapolar catheter described above.

**Cardiac O_2_ extraction** was estimated as arterio-venous difference in blood O_2_ content per 1L of blood, ie, *CaO_2_-CvO_2_*. Here *CaO*_2_ and *CvO_2_* is O_2_ content in arterial (coronary) and venous (coronary sinus) blood, respectively. *CaO_2_* (and similarly *CvO_2_*) is calculated as follows:

*CaO_2_ = (PaO_2_* x *α) + (SaO_2_* x *K* x *Hb)*,

where: *PaO_2_* is partial pressure of O_2_ in arterial blood (mmHg); *α* is O_2_ solubility coefficient (0.03 ml O_2_/1L blood/mmHg at 37°C); *SaO_2_* is arterial hemoglobin saturation; *K* is O_2_ capacity of fully saturated hemoglobin (1.38 mL O_2_/g Hb); *Hb* is hemoglobin concentration (g/L). *PaO_2_*, *SaO_2_* and *Hb* were obtained from blood samples as described above.

**IABP** was performed by a standard 30 cc IABP balloon (Arrow International Inc., Reading, PA, USA) insertion into the descending thoracic aorta and attachment to an Arrow IABP console; the 1:1 assist mode was tuned to the maximum obtainable diastolic augmentation. Internal mode set to 100 inflations/min was used during cardiac arrest with running ECMO.

**ECMO console, circuit and cannulation**

The ECMO circuit consisted of a MAQUET PLS and Rotaflow RF 32 centrifugal pump with HMO 70000 Adult Microporous Membrane Oxygenator with Softline Coating (MAQUET Cardiopulmonary AG, Hirrlingen, Germany), MAQUET PLS tubing set and a mechanical gas blender (Sechrist, Anaheim, CA, USA). Edwards cannulae (Fem-Flex Cannulae, Edwards Lifesciences Research Medical Inc., Midvale, UT, USA) were introduced percutaneously by the standard Seldinger technique. The venous inflow cannula was inserted via the femoral vein into the inferior vena cava just below the right atrium (the tip position was checked by fluoroscopy) and the femoral arterial outflow cannula was placed into the femoral artery. A subclavian artery access was obtained by a standard surgical cut-down and an open Seldinger technique. 19 or 21 French (F) cannulae were used for venous access and 15F and 17F cannulae were used to access the subclavian and femoral arteries, respectively. Constant circuit/animal temperature was regulated by the oxygenator heater unit HU 35 (MAQUET Cardiopulmonary AG, Hirrlingen, Germany) and kept normothermic within 36.5-37°C). Blood gases were monitored continuously in the arterial line of the extracorporeal circuit (CDI™ Blood Parameter Monitoring System 500, Terumo Cardiovascular Systems Corporation, Ann Arbor, MI, USA). The circuit, console function, and initial setting were controlled and directed by a perfusionist.

After connecting animals to ECMO, the fraction of delivered oxygen on the ECMO oxygenator gas blender was initially set to 40% and air flow to 2 L/min, and repeatedly adjusted according to continuously monitored arterial blood gases to maintain pO_2_ and pCO_2_ in ranges of 10 – 15 kPa and 4.5-5.6 kPa, respectively.
