# Supplementary figures and images for: Coronary versus carotid blood flow and coronary perfusion pressure in a pig model of prolonged cardiac arrest treated by different modes of venoarterial ECMO and intraaortic balloon counterpulsation
Source: Crit Care. 2012 Mar 16;16(2):R50. doi: 10.1186/cc11254 (PMC3964801; doi:10.1186/cc11254)

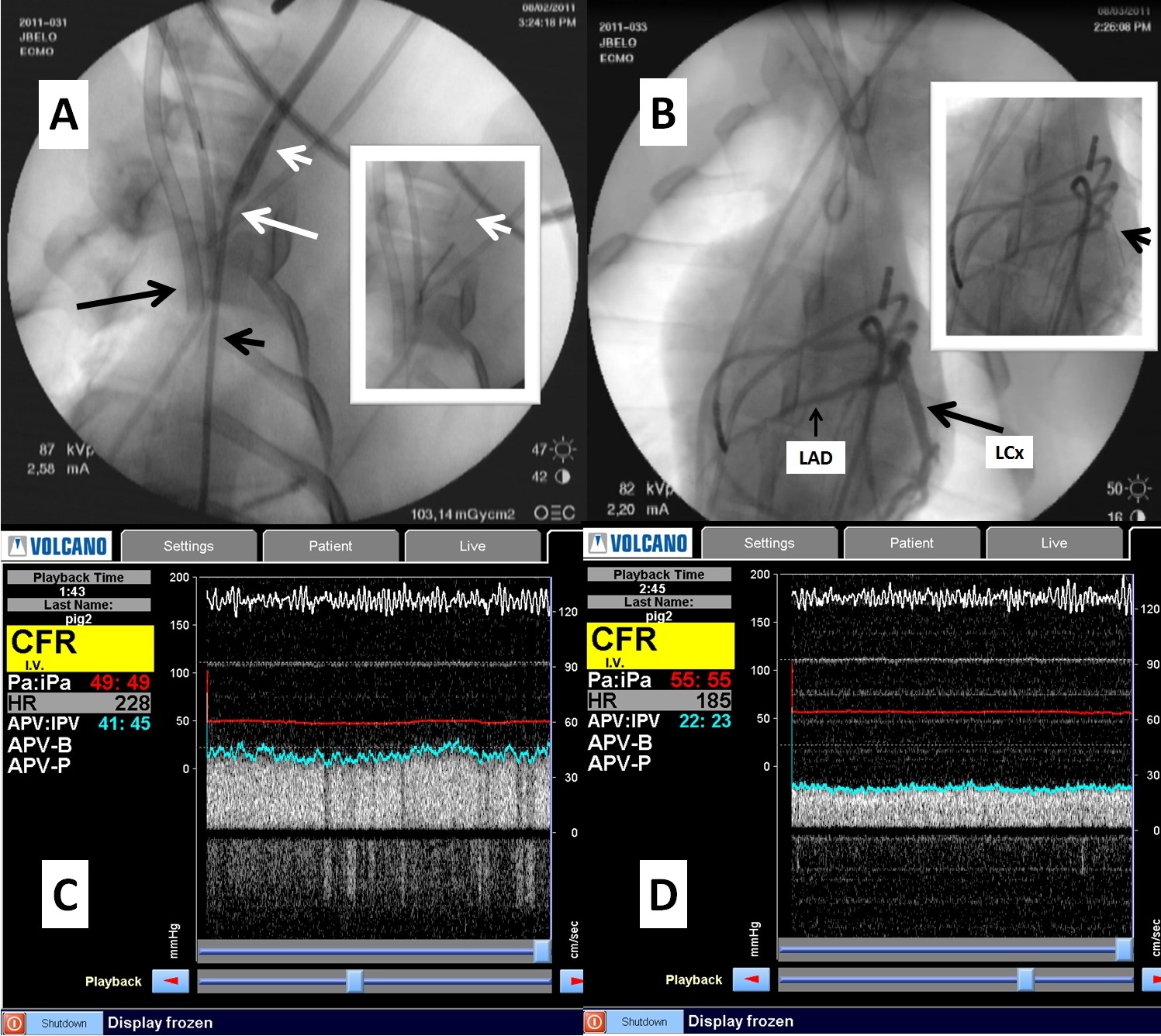

Supplement: Additional file 2 — Typical Doppler flow wire positions in carotid (panel A) and coronary (panel B) arteries. Panel A shows subclavian 15 French ECMO cannula (long black arrow), carotid angiography performed through carotid guiding catheter (short black arrow) positioned in the ostium of left carotid artery (long white arrow) and the tip of the Doppler flow wire (short white arrow), also shown in the box detail without contrast agent injection. Panel B shows coronary angiography with left anterior descending (LAD) (small black arrow) and left circumflex arteries (LCx) (long black arrow) and similarly in detailed box a tip of the Doppler wire in the proximal straight part of LCx (black arrowhead). At the bottom shown typical Doppler wire tracings obtained during cardiac arrest and running VA ECMO from carotid (panel C, APV = 41 cm/sec) and coronary (panel D, APV = 22 cm/sec) arteries. APV = average peak value. [file cc11254-S2.JPEG]

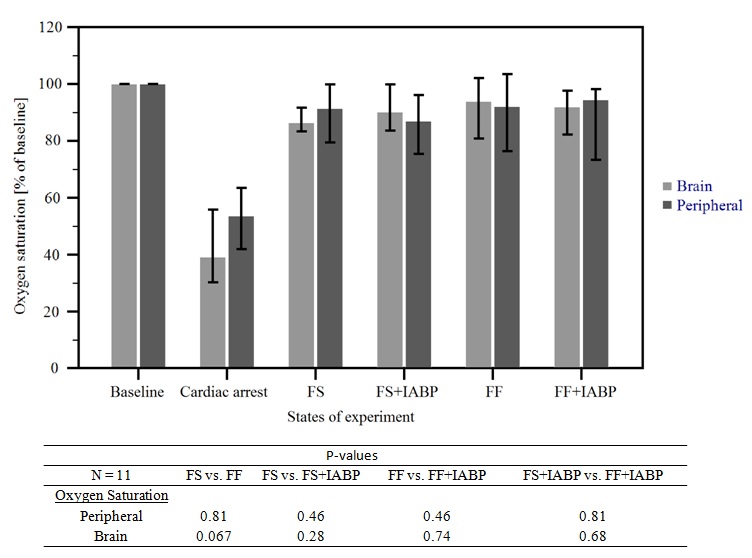

Supplement: Additional file 3 — Regional oxygen saturations measured by near infrared spectroscopy shown as percentage of baseline. After a dramatic decrease during cardiac arrest, sufficient restoration of both brain and peripheral saturations by all treatments is demonstrated. No significant difference for any of the comparisons noted. For actual values see Table 2. FS, femoro-subclavian; FF, femoro-femoral; IABP, intraaortic balloon counterpulsation. [file cc11254-S3.JPEG]

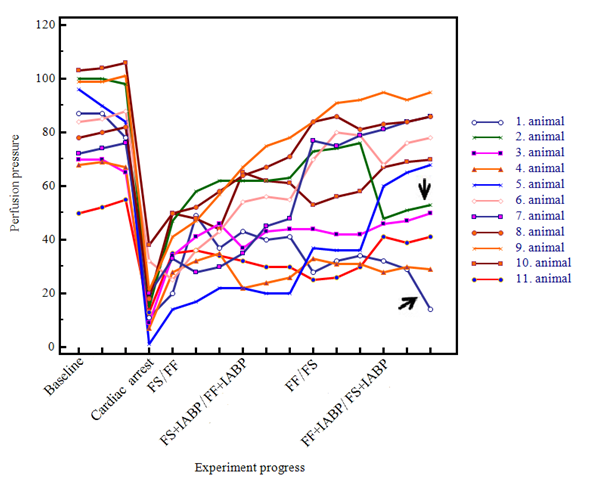

Supplement: Additional file 4 — Coronary perfusion pressure as measured in individual animals. The two animals with pressure drop (#1 and #2) marked by black arrows. [file cc11254-S4.TIFF]
