# Supplementary material for: Coronary versus carotid blood flow and coronary perfusion pressure in a pig model of prolonged cardiac arrest treated by different modes of venoarterial ECMO and intraaortic balloon counterpulsation
Source: Crit Care. 2012 Mar 16;16(2):R50. doi: 10.1186/cc11254 (PMC3964801; doi:10.1186/cc11254)
Supplement: Additional file 5 — Resuscitability of animals, number of defibrillations and ROSC presence (YES/NO) at 5 and 60 minutes. As vasopressors, epinephrine (Epi) and norepinephrine (Norepi) boluses followed by continuous IV drip were used only after defibrillations and CPR, doses given in mg for boluses and μg/kg/min for IV drip (norepinephrine). [file cc11254-S5.DOC]

| **Animal #** | **Number of defibrillations** | **Result after initial CPR** | **ROSC status** | | **Further interventions during post CPR period** | | |
| --- | --- | --- | --- | --- | --- | --- | --- |
|  |  |  | 5 min  (mmHg) | 60 min  (mmHg) | ECMO flow | Vasopressors | |
|  |  |  |  |  |  | Boluses  (mg) | IV drip - Norepi  (μg/kg/min) |
| 1 | 1 | PEA | NO (38) | NO (55) | ECMO switched off after defibrillation | NO | NO |
| 2 | 6 | VF | NO | NO | ECMO 5 L/min until min10, then switched off | NO | NO |
| 3 | 2 | SV rhythm | NO (58) | YES (67) | ECMO 5 L/min until min 60 | NO | 0.4 |
| 4 | 1 | SV rhythm | YES (70) | YES (88) | ECMO ↓ 3 L/min at min 10, thereafter 3-4 L/min until min 60 | Norepi 1 | 0.06 |
| 5 | 1 | SV rhythm | YES (71) | YES (78) | ECMO ↓ 0,4 L/min after CPR, switched off at min 45 | NO | NO |
| 6 | 1 | PEA | YES (67) | YES (84) | ECMO ↓ 3,6 L/min at min 30, switched off at min 50 | Epi 1 + Norepi 0.7 | 0.2 |
| 7 | 1 | SV rhythm | YES (99) | YES (90) | ECMO ↓ 2 L/min at min 5 until min 60 | NO | NO |
| 8 | 1 | SV rhythm | YES (88) | YES (60) | ECMO 3-4 L/min until min 60 | NO | NO |
| 9 | 1 | PEA | YES (82) | YES (83) | ECMO ↓ 0,4 L/min at min 10 until min 60 | Epi 1 | NO |
| 10 | 1 | SV rhythm | YES (87) | YES (69) | ECMO ↓ to 1,3 L/min at min 10 and 0,3 L/min at min 35, switched off at min 40. | Epi 1 | NO |
| 11 | 1 | SV rhythm | YES (76) | NO (27) | ECMO ↓ to 3,2 L/min at min 10 and kept 2,5-3,8 L/min until min 60 | Epi 1 | 0.3 to 0.4 |

**Abbreviations:** CPR= cardiopulmonary resuscitation; ECMO= extracorporeal membrane oxygenation; Epi= epinephrine; IV= intravenous; Min= minute; Norepi= norepinephrine; PEA= pulseless electrical activity;ROSC= return of spontaneous circulation; SV= supraventricular; VF= ventricular fibrillation; ↓= decrease
